# Current Knowledge and Novel Frontiers in Lower Urinary Tract Dysfunction after Spinal Cord Injury: Basic Research Perspectives

**DOI:** 10.4103/uros.uros_31_22

**Published:** 2022-08-25

**Authors:** Naoki Wada, Sergei Karnup, Katsumi Kadekawa, Nobutaka Shimizu, Joonbeom Kwon, Takahiro Shimizu, Daisuke Gotoh, Hidehiro Kakizaki, William C. de Groat, Naoki Yoshimura

**Affiliations:** 1 Department of Urology, University of Pittsburgh School of Medicine, Pittsburgh, PA, USA; 2 Department of Renal and Urologic Surgery, Asahikawa Medical University, Asahikawa, Japan; 3 Department of Pharmacology and Chemical Biology, University of Pittsburgh School of Medicine, Pittsburgh, PA, USA

**Keywords:** Brain-derived neurotrophic factor, detrusor overactivity, detrusor-sphincter dyssynergia, lower urinary tract, nerve growth factor, spinal cord

## Abstract

This review article aims to summarize the recent advancement in basic research on lower urinary tract dysfunction (LUTD) following spinal cord injury (SCI) above the sacral level. We particularly focused on the neurophysiologic mechanisms controlling the lower urinary tract (LUT) function and the SCI-induced changes in micturition control in animal models of SCI. The LUT has two main functions, the storage and voiding of urine, that are regulated by a complex neural control system. This neural system coordinates the activity of two functional units in the LUT: the urinary bladder and an outlet including bladder neck, urethra, and striated muscles of the pelvic floor. During the storage phase, the outlet is closed and the bladder is quiescent to maintain a low intravesical pressure and continence, and during the voiding phase, the outlet relaxes and the bladder contracts to promote efficient release of urine. SCI impairs voluntary control of voiding as well as the normal reflex pathways that coordinate bladder and sphincter function. Following SCI, the bladder is initially areflexic but then becomes hyperreflexic due to the emergence of a spinal micturition reflex pathway. However, the bladder does not empty efficiently because coordination between the bladder and urethral sphincter is lost. In animal models of SCI, hyperexcitability of silent C-fiber bladder afferents is a major pathophysiological basis of neurogenic LUTD, especially detrusor overactivity. Reflex plasticity is associated with changes in the properties of neuropeptides, neurotrophic factors, or chemical receptors of afferent neurons. Not only C-fiber but also Aδ-fiber could be involved in the emergence of neurogenic LUTD such as detrusor sphincter dyssynergia following SCI. Animal research using disease models helps us to detect the different contributing factors for LUTD due to SCI and to find potential targets for new treatments.

## Introduction

The lower urinary tract (LUT) stores and periodically releases urine. These functions depend on neural circuits located in the brain, spinal cord, and peripheral ganglia.^[1,2]^ Spinal cord injury (SCI) rostral to the lumbosacral level eliminates voluntary and supraspinal control of voiding, inducing initially an areflexic bladder leading to urinary retention. Later, neurogenic detrusor overactivity (DO) develops along with loss of detrusor and urethral sphincter coordination (termed detrusor sphincter dyssynergia or DSD) resulting in inefficient voiding, bladder hypertrophy, and high intravesical pressure. The recovery of reflex bladder activity after SCI is dependent on the reorganization of reflex pathways in the spinal cord and alterations in the properties of bladder afferent neurons.^[3,4]^ Hence, the clinical management of SCI patients should focus on LUT dysfunction (LUTD) during the storage phase (DO and LUTD) and the voiding phase (DSD and inefficient voiding). Previous studies including ours have attempted to elucidate the neurologic mechanisms crucial for the diagnosis and treatment of LUTD after SCI using SCI animal models. In this article, we focus on the neurophysiologic mechanisms involved in the control of LUT function and the changes in micturition control induced after SCI above the sacral level. We have also summarized the latest basic research studies identifying targets for treating LUTD following SCI using animal models.

## Neurophysiology of the Lower Urinary Tract

### Bladder

The LUT consists of the bladder and the urethra that store and eliminates urine, respectively. These structures are regulated by three sets of peripheral nerves: sacral parasympathetic (pelvic), thoracolumbar sympathetic (hypogastric and sympathetic chain), and somatic (pudendal) nerves distributed bilaterally,^[2,5]^ which consist of efferent and afferent axons originating at thoracolumbar and sacral spinal levels.^[4]^ As the bladder fills during the storage phase, the detrusor remains quiescent with minor changes in intravesical pressure. The neural pathways stimulating the bladder for micturition are quiescent during this phase because of the active inhibitory pathways.^[2,6,7]^

Although traditionally considered a passive barrier, the bladder urothelium also has specialized sensory and signaling properties that make it responsive to chemical and mechanical stimuli and engage in reciprocal chemical communication with neighboring nerves or myofibroblasts in the underlying lamina propria, which in combination with the urothelial layer comprises the bladder mucosa.^[8–10]^ The urothelium exhibits many properties including the following: (1) expression of acetylcholine (ACh), norepinephrine (NE), tachykinins, and agonists for transient receptor potential (TRP) channels (TRPV1, TRPV4, TRPM8 receptors); (2) close physical association with afferent nerves; and (3) ability to release chemical mediators, such as adenosine triphosphate (ATP), ACh,^[4]^ nerve growth factor (NGF), and nitric oxide (NO),^[11–13]^ that can regulate the activity of adjacent nerves or myofibroblasts and thereby influence reflex bladder contractions.^[9,14]^

### External urethral sphincter

**Figure 1:**
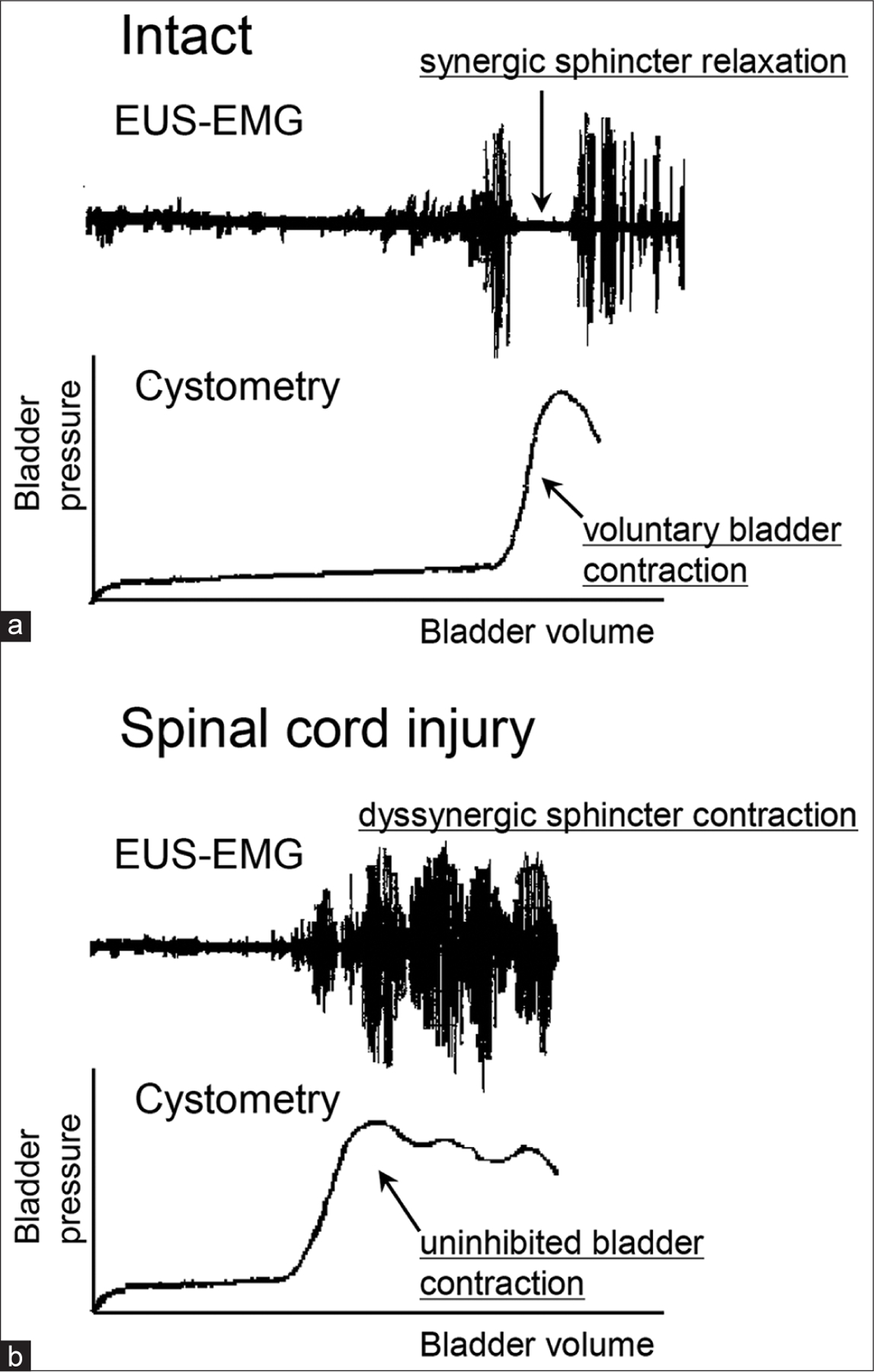
Representative CMG and EUS-EMG recording in human. (a) CMG and EUS-EMG recordings under the normal condition show that synergic urethral relaxation (inactive EUS-EMG) occurs when voluntary bladder contraction is induced. (b) CMG and EUS-EMG after spinal cord injury show uninhibited bladder contractions (DO and DSD). CMG: Cystometrogram, EUS: External urethral sphincter, EMG: Electromyogram, DO: Detrusor overactivity, DSD: Detrusor-sphincter dyssynergia

**Figure 2:**
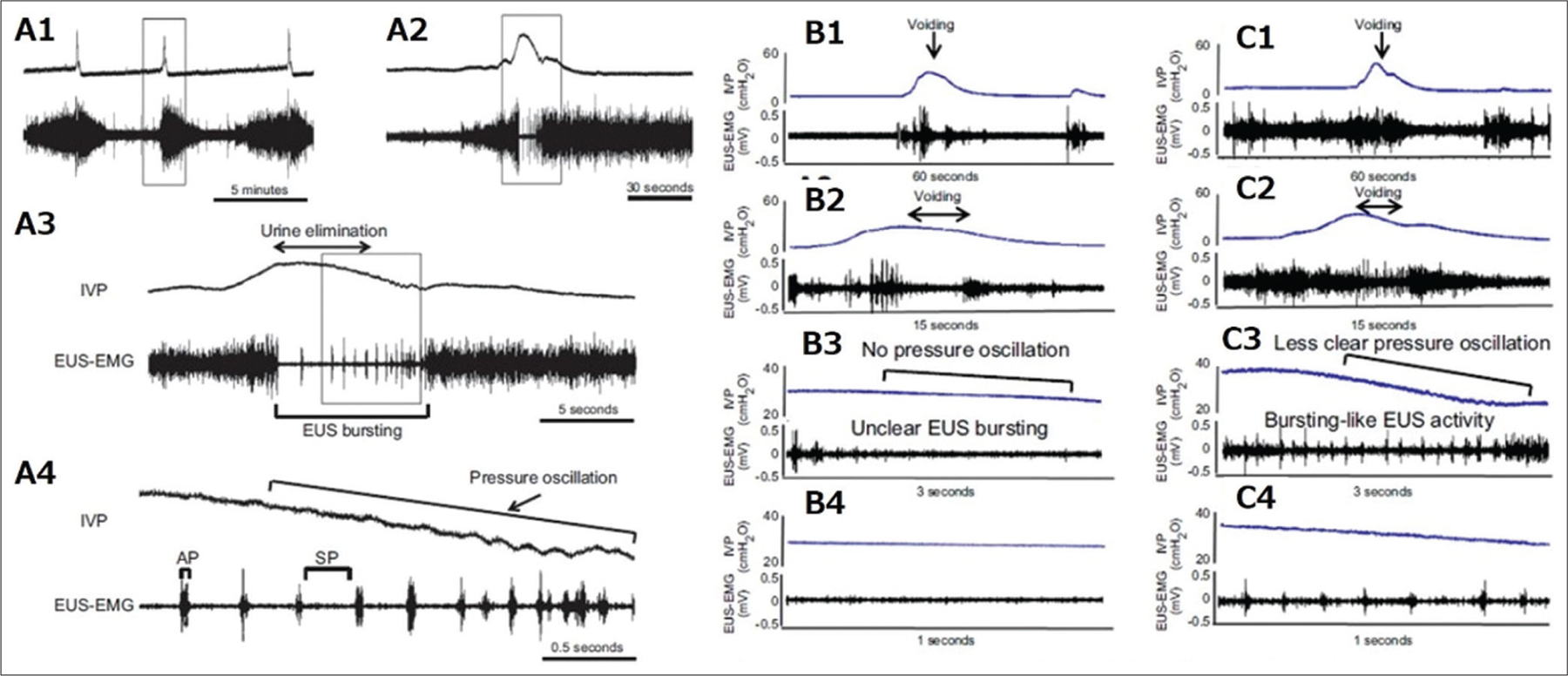
Representative recordings of simultaneous measurement of CMG and EUS- EMG activity in spinal intact unanesthetized rodents. Traces A2–4, B2–4, and C2–3 depict sections of traces A1, B1, and C1, respectively, on expanded time scales. A1–A4 show the IVP and EUS-EMG recordings during a continuous infusion CMG in a spinal intact rat. The EUS-EMG exhibits tonic activity before the onset of voiding and bursting activity during voiding (A2–4). The EUS bursting is characterized by clusters of high-frequency spikes (APs) separated by low tonic activity (SPs) (A3 and A4). The bursting produces rhythmic contractions and relaxations of the EUS and is thought to generate a urethral pumping action during voiding, which is seen as pressure oscillations on the continuous CMG tracing (A4). B1–B4 and C1–C4 show the IVP and EUS-EMG recordings during a continuous infusion CMG in a spinal intact mouse without pressure oscillation (B1–B4), and in a spinal intact mouse with pressure oscillation (C1–C4). Some mice exhibit reduced EUS activity without bursting and no obvious pressure oscillation during voiding (B1–B4). The other mice exhibit bursting-like EUS activity during voiding bladder contractions, similar to EUS busting in spinal intact rats (C1–C4), consisting of APs, and reduced EMG activity (SPs), which coincide with IVP oscillations in the CMG tracing, but the IVP oscillations in the CMG tracing were less obvious compared with those in spinal intact rats. (Modified from Kadekawa *et al*., 2016^[19]^). CMG: Cystometrogram, EUS: External urethral sphincter, EMG: Electromyogram, IVP: intravesical pressure, Aps: Active periods, SPs: Silent periods, Aps: Alternating tonics

External urethral sphincter (EUS)-electromyogram (EMG) recordings are widely used for evaluating urethral function. In various species including humans, the EUS contracts (active EUS-EMG phase) to maintain urinary continence during the storage phase and relaxes (inactive EUS-EMG phase) to allow urine flow through the urethra during the voiding phase [Figure 1]. However, in some species, such as healthy rats or dogs, EUS-EMG recordings exhibit tonic activity before the onset of voiding and bursting activity during voiding [Figure 2]. This phenomenon is characterized by clusters of high-frequency spikes (active periods) separated by low-tonic activity (silent periods [SPs]), producing a pumping action (rhythmic contractions and relaxations of EUS), which is seen as intravesical pressure oscillations during cystometrograms (CMGs).^**[**2,15–19]^ The EUS bursting activity and pressure oscillations in CMGs are abolished by a neuromuscular blocker (bungarotoxin) or pudendal nerve transection in rats, suggesting that the EUS-pumping activity is important for efficient bladder emptying.^[17,20]^ Furthermore, recent studies in rats demonstrated that coordination of EUS activity and bladder during voiding is dependent on neurons in the L3/L4 spinal cord, and this L3/L4 EUS-related circuitry consists of at least two neuronal populations: segmental interneurons and propriospinal neurons projecting to the L6/S1 spinal cord, where the major output signals carried through the pudendal nerve from the Onuf’s nucleus to EUS originate.^[21–24]^ Contrastingly, the EUS-pumping activity during voiding does not occur in humans, and only a minority of healthy female mice exhibit EUS bursting activity with little impact on voiding efficiency [Figure 2].^[19]^

### Efferent nerves

The LUT efferent pathways are innervated by parasympathetic, sympathetic, and somatic nerves with the parasympathetic pathway providing the major excitatory input to the bladder.^[2,7]^ Cholinergic parasympathetic preganglionic neurons located in the intermediolateral region of the sacral spinal cord send axons through the pelvic nerves to the ganglion cells in the pelvic plexus and the bladder wall.^[5]^ Parasympathetic postganglionic nerves excite the bladder smooth muscles by releasing cholinergic (ACh) and nonadrenergic, noncholinergic transmitters (ATP) that act on muscarinic cholinoceptors and P2X1 purinoceptors, respectively.^[5]^ Postganglionic axons continue for a short distance in the pelvic nerve and terminate in the detrusor layer, where they release ACh to induce contractions of the detrusor smooth muscle fibers. Muscarinic inhibitory and facilitatory receptors are also present on parasympathetic nerve terminals.^[25]^ Along with parasympathetic stimulation of bladder smooth muscles, some postsynaptic parasympathetic neurons cause relaxation of the urethral smooth muscle, possibly through NO release.^[26–28]^

Sympathetic pathways to the LUT, originating in the lumbar spinal cord, elicit various effects, including the following: (1) inhibition of the detrusor muscle through β-adrenoceptors and (2) excitation of the bladder neck and urethra through α -adrenoceptors.^[2,5]^ Preganglionic sympathetic neurons are located in the intermediolateral column of thoracolumbar cord segments T11 to L2 in humans and^[26]^ most preganglionic fibers synapse with postganglionic neurons in the inferior mesenteric ganglia. The preganglionic neurotransmitter, ACh, acts through nicotinic receptors in the postganglionic neurons whose axons travel in the hypogastric nerve and release NE at their terminals. Somatic nerves provide excitatory innervation to the striated muscles of the EUS and pelvic floor. The efferent motoneurons are in the Onuf’s nucleus, along the lateral border of the ventral horn in sacral spinal cord segments S2 to S4 in humans.^[29]^ Motoneuron axons are present in the pudendal nerve and release ACh at their terminals, which acts on nicotinic receptors in the striated muscle, inducing muscle contraction to maintain EUS closure.

### Afferent nerves

Afferent fibers of the pelvic, hypogastric, and pudendal nerves carry sensory information from the LUT to the lumbosacral region.^[2,4,30]^ The afferents passing from the pelvic nerve to the sacral spinal cord are important for initiating micturition. Light and electron microscopy studies have revealed that the visceral nerves innervating the LUT are composed primarily of small myelinated (Aδ-fiber) and unmyelinated (C-fiber) axons.^[31,32]^ Dorsal root ganglion (DRG) neurons give rise to myelinated Aδ- and unmyelinated C-fiber axons, which are distinguishable through immunohistochemical staining for a 200 kDa neurofilament (NF) protein that is exclusively expressed in the myelinated Aδ-fiber DRG neurons.^[33]^ Approximately two-thirds of the bladder afferent neurons identified in rats using axonal tracing methods are NF-poor (i.e., C-fiber neurons), which are sensitive to capsaicin. The remaining neurons exhibit intense NF immunoreactivity (Aδ-fiber neurons).^[34]^ These afferents, including small myelinated (Aδ) and unmyelinated (C) fibers, convey information from receptors in the bladder wall to second-order neurons in the spinal cord. In cats, the Aδ bladder afferents respond in a graded manner to passive distension and active contraction of the bladder, whereas the unmyelinated C-fiber ones are insensitive to mechanical stimuli and do not respond to even high levels of intravesical pressure.^[4,35]^ Silent C-fiber afferents have a specialized function, such as signaling during inflammatory or noxious events in the LUT.

## Changes in Micturition Control Induced After Spinal Cord Injury

### Changes in the bladder

In animals and humans with suprasacral lesions, CMGs exhibit intrinsic and reflex contractions (i.e., neurogenic DO) during bladder filling that are absent in individuals with intact spinal cords.^[1,3,36]^ In addition, maximal voiding pressure is increased, voiding efficiency is reduced, and the bladder undergoes marked hypertrophy.^[15,16,37]^ Recordings of contractile activity of *in vitro* whole bladders or bladder strips from SCI animals reveal large-amplitude phasic contractions similar to those seen in adult bladders with partial outlet obstruction.^[38–42]^

The bladder urothelium is also altered after SCI in rodents. In rats, within 2 h after spinal transection, the umbrella cell layer continuity is disrupted, and the transepithelial resistance (TER) is markedly reduced.^[43]^ Twenty-four hours after SCI, TER reaches a minimum, and water and urea permeability are significantly increased. Although these changes are reversed within 2 weeks, they can be prevented by treatment with a ganglionic blocking agent, indicating that they are mediated by activity in autonomic nerves. Long-term changes include increased the expression of gap junction proteins: connexin 26 in the urothelium and connexin 43 in the lamina propria in rat bladders,^[38]^ altering bladder function after SCI. Optical imaging techniques have revealed that phasic contractions in SCI mice bladders originate from localized sites in the bladder dome and are driven by activity arising in the urothelium.^[44]^ Removal of the mucosa eliminated these contractions in SCI rat bladders.^[39]^ In addition, gap junction blockers suppress phasic contractions.^[38]^ On the basis of this, it was proposed that phasic activity in bladders after SCI might be due to signaling pathways originating in the urothelium and then passing through gap junctions through the myofibroblast network in the lamina propria to smooth muscles and afferent nerves.^[39]^

### Changes in the external urethral sphincter

**Figure 3:**
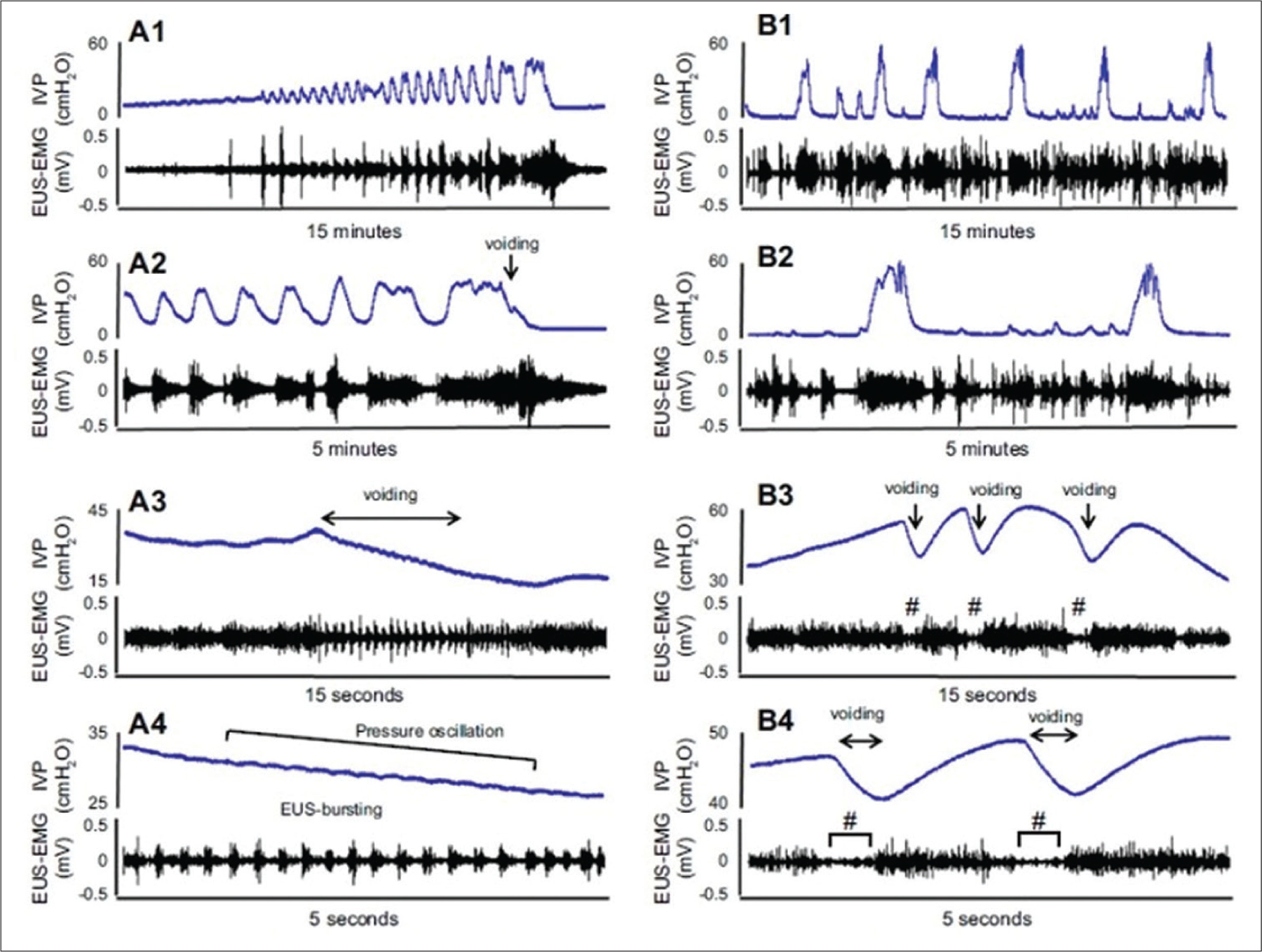
Representative recordings of simultaneous measurement of CMG and EUS- EMG activity in a SCI rat (A1–A4) and an SCI mouse (B1–B4). Traces A2–A4 and B2–B4 are expanded from the traces A1 and B1, respectively, with different time scales. The SCI rat (A1–A4) exhibited EUS bursting with alternating APs and SPs during voiding bladder contractions, which coincided with rapid pressure oscillations in the CMG tracing. The SCI mouse (B1–B4) had intermittent voiding coinciding with more prolonged reductions in intravesical pressure in the CMG recording, which occurred during periods of low-tonic, synergic EUS-EMG activity (B3 and B4). Clear EUS bursting activity or intravesical pressure oscillations were not seen in the SCI mouse. #symbols in B3 and B4 indicate the reduced EUS activity (R-EA) during voiding.(Modified from Kadekawa *et al*., 2016^[19]^). CMG: Cystometrogram, EUS: External urethral sphincter, EMG: Electromyogram, SCI: Spinal cord-injured

Although DO during urine storage is similar in both rats and mice after SCI,^[44,45]^ the behavior of the EUS during voiding is different in these two species.^[19]^ In SCI rats, EUS bursting occurs during voiding bladder contractions, coinciding with small amplitude intravesical pressure oscillations during cystometry [Figure 3]. Nevertheless, instead of clear EUS bursting or intravesical pressure oscillation, SCI mice exhibit intermittent voiding with slow, large amplitude reductions in intravesical pressure, occurring during periods of reduced EUS activity [Figure 3].^[19]^ α-bungarotoxin improves voiding by reducing urethral outlet resistance in SCI rats.^[20]^ However, in healthy rats, it reduces voiding efficiency by suppressing high-frequency phasic sphincter activity necessary for efficient urine elimination in spinal intact conditions. Although the reflex EUS-pumping activity recovers and promotes voiding in rats after SCI, it is not seen in female mice.^[19]^ This might be because of the difference in urethral activity during the voiding phase in these two species. Bilateral transection of the hypogastric nerves, which provide the major sympathetic input to the urinary bladder and proximal urethra, improves voiding in SCI rats, suggesting that abnormal sympathetic reflexes are also involved in LUTD after SCI.^[46]^ In this article, we focus on SCI-induced DSD resulting in inefficient voiding, which is the major urethral dysfunction after SCI. Previous studies have shown that α-adrenoceptor blockers effectively improved SCI-induced inefficient voiding by reducing urethral pressure in rats,^[47,48]^ although they suggested that their effects are mediated by changes in EUS function or improvement of bladder ischemia. Nevertheless, the functional changes in the internal urethral sphincter comprising smooth muscles after SCI must be further explored.

### Changes in bladder reflexes

Studies show that C-fiber bladder afferents, which usually do not respond to bladder distention (i.e., silent C-fibers), become mechanosensitive and initiate automatic micturition after SCI.^[35]^ In SCI cats, micturition is induced by C-fiber afferent pathways. In chronic spinalized cats, desensitization of TRPV1-expressing C-fiber afferent pathways by subcutaneous administration of capsaicin, a C-fiber neurotoxin that binds to TRPV1 receptors, can significantly block DO during the storage phase, whereas this inhibitory effect is not seen on reflex bladder contractions in spinal intact cats.^[49,50]^ In SCI rats, both Aδ and C-fiber afferents can evoke bladder reflexes^[36]^ and increased excitability of the latter induces DO because desensitizing C-fiber afferents using systemic capsaicin administration substantially suppressed nonvoiding bladder contractions without affecting the voiding reflex.^[51]^ These results indicate that C-fiber afferents are necessary for generating neurogenic DO during the storage phase, whereas Aδ afferents still initiate voiding in SCI rats.

Capsaicin-sensitive C-fiber bladder afferent pathways, which become hyperexcitable after SCI, are involved in the control of bladder activity in both spinal intact and SCI mice. In these mice, DO is significantly but partially dependent on capsaicin-sensitive C-fiber afferents in contrast to SCI rats,^[52]^ in which DO is induced predominantly by capsaicin-sensitive C-fiber afferents.^[51]^ Thus, mice and rats seem to have different C-fiber-dependent afferent mechanisms controlling bladder function.

In humans with neurogenic DO, intravesical administration of C-fiber neurotoxins (capsaicin or resiniferatoxin) symptomatically improves and reduces the density of TRPV1 and P2X3 immunoreactive nerve fibers and urothelial TRPV1 immunoreactivity.^[1,53,54]^ Randomized controlled trials with SCI patients revealed that these neurotoxins effectively improved both urodynamic and clinical parameters.^[1]^ Injecting botulinum neurotoxin type A, an agent that blocks neurotransmitter release from urothelial cells and from afferent and efferent nerves, into the bladder wall also reduces neurogenic DO and reduces the density of TRPV1- and P2X3-immunoreactive nerves.^[55]^ These findings suggest that abnormal C-fiber afferent innervation contributes to neurogenic DO in SCI humans.

## Plasticity of Neurotransmitter Mechanisms

### Neurokinins

Destruction of lumbosacral spinal neurons expressing neurokinin-1 receptors (NK-1R) using an NK-1 ligand conjugated with saporin does not affect the voiding reflex in rats with intact spinal cords but reduces the bladder irritant effects of intravesical capsaicin and reduces nonvoiding contractions in SCI rats.^[56,57]^ Similarly, intrathecal administration of a selective NK-1R antagonist does not affect the micturition reflex in spinal intact rats but blocks it in SCI rats.^[58]^ These data, coupled with the increased substance *P* expression in the sacral parasympathetic nucleus region in SCI rats, indicate that NK-1R activation is involved in micturition in paraplegic animals.^[58]^

NK-2R agonists act as bladder prokinetic agents and induce smooth muscle contractions by stimulating NK-2R in smooth muscle cells.^[59–61]^ NK-2R agonists can produce rapid-onset and short-duration, dose-dependent elevations in bladder pressure.^[62–64]^ In conscious, spinal intact dogs, NK-2R agonists elicit efficient voiding within minutes of the administration.^[64]^ This effect is also observed in anesthetized, acute SCI rats.^[62]^ Chronic administration of NK-2R agonists produces consistent and efficient voiding in SCI rats.^[65]^ Thus, NK receptors might act as therapeutic targets for the treatment of SCI-related LUT dysfunction.

### Gamma-aminobutyric acid

In the central nervous system, glutamate is a major excitatory amino acid, whereas glycine and gamma-aminobutyric acid (GABA) are major inhibitory neurotransmitters that inhibit the micturition reflex at supraspinal and/or spinal sites with additive or synergistic inhibitory effects on bladder activity.^[66]^ Intrathecal, intravenous, or dietary glycine inhibits both bladder and urethral activity in normal and SCI rats.^[67,68]^ Hypofunction of glycinergic or GABAergic mechanisms in the lumbosacral spinal cord induces LUTD, such as DO or DSD, in SCI rats.^[18,67,69]^ Furthermore, intrathecal muscimol and baclofen (GABA_A_ and GABA_B_ agonists, respectively) inhibit nonvoiding bladder contractions by suppressing C-fiber bladder afferents and improve DSD in SCI rats.^[18,69]^ Reduced GABAergic inhibition might contribute to the development of neurogenic DO because mRNA levels of glutamic acid decarboxylase (GAD), an enzyme involved in GABA synthesis, are decreased in the spinal cord after SCI.^[69]^ Gene delivery of GAD using nonreplicating herpes simplex virus (HSV) vectors inhibits DO or DSD without affecting the voiding contraction in SCI rats.^[70,71]^

### Role of neurotrophic factors

#### Nerve growth factor

**Figure 4:**
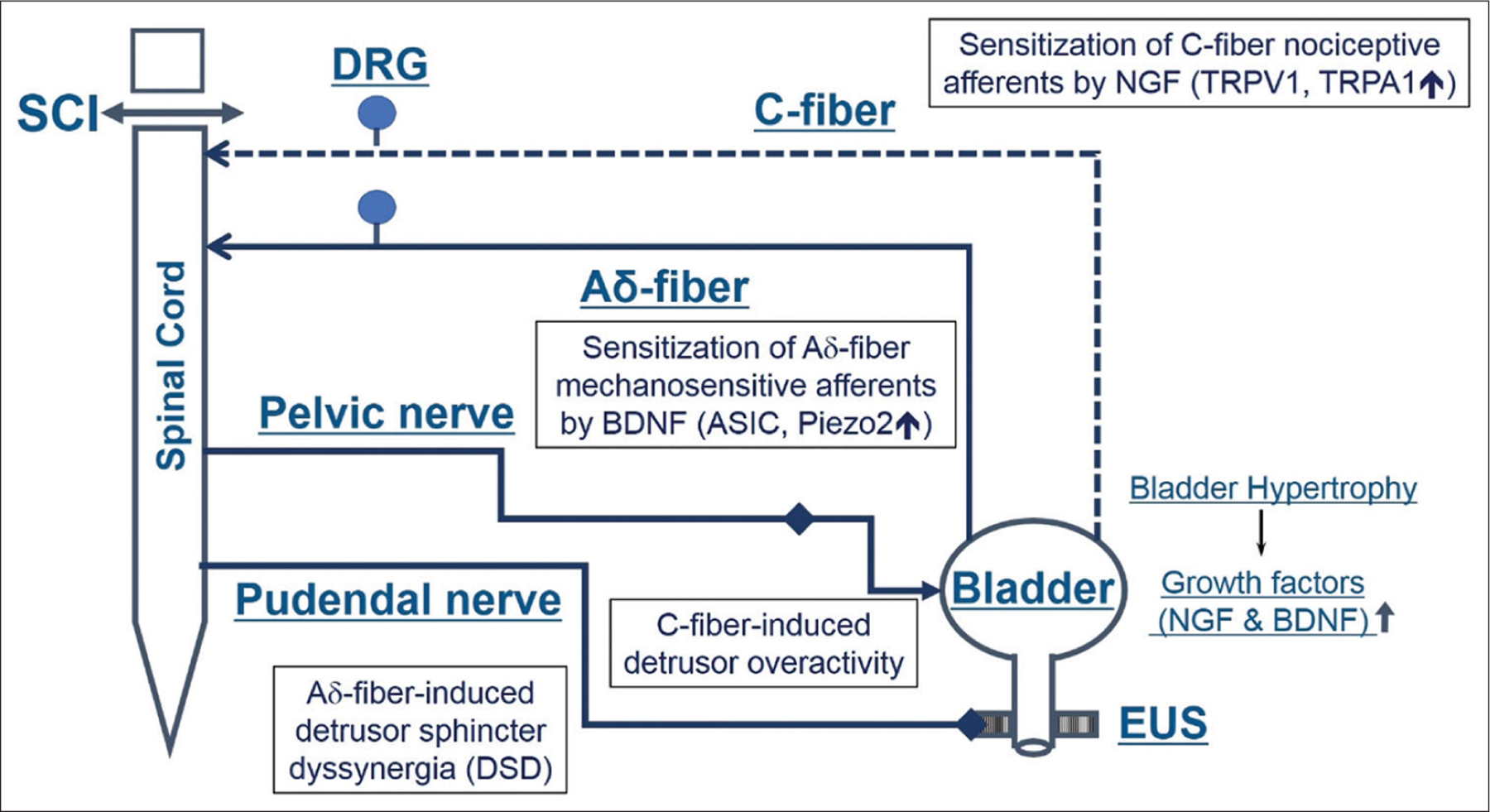
Diagram showing hypothetical mechanisms underlying storage and voiding dysfunction induced by increasing expression of neurotrophic factors following SCI. Injury to the spinal cord causes DSD, leading to functional urethral obstruction, reduced voiding efficiency, urinary retention and bladder hypertrophy, resulting in increased levels of NGF in the bladder. NGF is taken up by afferent nerves and transported to the DRG cells. The levels of NGF also increase in the spinal cord after SCI. TrkA, which responds to NGF, is abundant in bladder afferents, especially C-fiber neurons. Hyperexcitability of bladder C-fiber afferent pathways causes or enhances neurogenic DO. BDNF is also increased in the bladder and the spinal cord after SCI. TrkB, which responds to BDNF, is expressed on larger-sized bladder afferent neurons, presumably Aδ-fiber, that express mechanosensitive receptors, ASIC and Piezo 2. Hyperexcitability of bladder Aδ-fiber afferent pathways causes or enhances DSD. Systemic application of BDNF antibodies reduces BDNF levels in the spinal cord and improves DSD. SCI: Spinal cord injury, DSD: Detrusor-sphincter dyssynergia, NGF: Nerve growth factor, DRG: Dorsal root ganglion, DO: Detrusor overactivity, BDNF: Brain derived neurotrophic factor, TrkB: Tropomyosin receptor kinase B

Neurotrophic factors including NGF, brain-derived neurotrophic factor (BDNF), NT-3, and NT-4 are important in various types of neural plasticity including the emergence of LUTD in SCI.^[13,72–74]^ NGF is a well-researched neurotrophin that regulates the developing nervous system. DSD-induced overdistention of the bladder and decreased voiding efficiency after SCI increases NGF production in the bladder that might enhance C-fiber afferent nerve excitability, leading to neurogenic DO [Figure 4]^[73,75–77]^ because capsaicin treatment inhibits bladder overactivity without affecting voiding contractions in SCI rats and mice.^[36,51,52]^ Both neutralization of NGF and desensitization of C-fiber afferents with capsaicin or resiniferatoxin similarly suppress bladder overactivity in SCI rats and mice.^[52,75,77,78]^ NGF production is increased not only in the bladder but also in the lumbosacral spinal cord and in DRG.^[77,79]^ Overdistension of the bladder might stimulate an increase in NGF levels because its levels also increase in the bladder of rats after partial obstruction of the urethral outlet or in the urine of humans with bladder outlet obstruction.^[80,81]^ Reports have shown that NGF produced in the bladder is transported to DRG cell bodies and their spinal projections.^[13]^ Intravesical administration of NGF in rats acutely increases reflex bladder activity, and chronic administration into the spinal cord or the bladder wall of rats induces bladder hyperactivity and increases the firing frequency of dissociated bladder afferent neurons.^[73,79,82,83]^

The immunoreactivity of TrkA, a high-affinity NGF receptor that is predominantly expressed in C-fiber afferent neurons, is increased in bladder afferent neurons of SCI rats.^[84,85]^ The NGF-TrkA axis is one of the important regulators of TRPV1 expression, spatial distribution, and activation thresholds in sensory neurons.^[86,87]^ A novel HSV vector-mediated neuronal labeling technique indicated that SCI induces expansion of the TRPV1-expressing C-fiber cell population, which might contribute to C-fiber afferent hyperexcitability.^[88]^ TRPV1 upregulation is closely related to NGF overexpression after SCI. The systemic administration of TRPV1 or TRPA1 antagonists reduced both bladder contraction frequency and bladder hyperactivity.^[89,90]^ NGF neutralization downregulates NGF levels both at the bladder and the spinal cord and inhibits bladder hyperactivity along with reduced TRPV1 and TRPA1 expression in L6-S1 DRG.^[77]^ Taken together, NGF is probably produced in the bladder after SCI, contributing to C-fiber afferent hyperexcitability mediated through TRP channels including TRPV1 and TRPA1, inducing the emergence of bladder overactivity.

#### Brain-derived neurotrophic factor

BDNF levels in the bladder and spinal cord are upregulated as early as 4–5 days after SCI in rats and mice.^[84,91,92]^ BDNF induces various physiological and pathological changes by acting mainly on TrkB, a high-affinity, ligand-specific receptor of BDNF found in many neurons in the spinal cord and primary afferent pathways.^[93]^ Although TrkB is expressed in both large (Aδ-fiber) and small (C-fiber) primary afferent neurons, higher TrkB expression is seen in large-sized, Aδ-fiber bladder afferent neurons [Figure 4].^[85,94]^

In both SCI rats and mice, the afferent limb of micturition reflexes inducing bladder overactivity possibly consists of C-fiber afferents because capsaicin pretreatment significantly reduced bladder hyperactivity in both species.^[51,52]^ Previous studies reported that BDNF inhibition improved voiding function in chronic SCI rats whereas BDNF inhibition initiated during the post-SCI spinal shock phase resulted in the enhanced bladder overactivity, suggesting that BDNF in the early phase of SCI contributes to C-fiber-mediated bladder overactivity.^[92,95]^

BDNF neutralization in 4-week-old SCI mice increases voided volume and voiding efficiency.^[91,92]^ In SCI mice, periodic reductions of EUS-EMG activity are observed during voiding bladder contractions, which coincides with notch-like reductions in intravesical pressure, reflected as urethral synergic relaxation that promotes voiding [Figure 3].^[19]^ The durations of reduced EUS-EMG and notch periods are increased after BDNF neutralization, indicating improved urethral synergic relaxation during the voiding phase in SCI mice.^[91,92]^

Acid-sensing ion channels (ASICs) expressed by sensory neurons might also be a molecular target of BDNF in bladder afferent pathways. Although ASICs were originally identified as receptors activated in response to diminished extracellular pH,^[96,97]^ they were later found to also have mechanosensory functions.^[98]^ ASIC3 receptors are expressed in TRPV1-expressing, unmyelinated C-fiber neurons and in mechanosensitive myelinated A-fiber neurons in DRG.^[99,100]^ The ASIC2 channel, which is also a BDNF signaling target, regulates sensory mechanotransduction.^[97]^ In the recent study, ASIC2 and ASIC3 transcripts in L6-S1 DRG were upregulated in SCI mice, which were suppressed after BDNF neutralization. By contrast, TRPV1 expression in L6-S1 DRG, which was increased after SCI, was not altered after BDNF neutralization.^[91]^ Thus, BDNF might increase mechanotransduction through Aδ-fiber bladder afferent pathways to enhance the bladder-to-EUS reflex, leading to DSD and inefficient voiding after SCI [Figure 4].

#### Targeting p75 neurotrophin receptors

Mature BDNF and NGF are enzymatically processed within secretory vesicles from their proneurotrophins (proBDNF/proNGF) before release.^[101]^ Under pathological conditions, their overexpression exceeds the processing rate leading to the unregulated release of proneurotrophins, which differ from mature forms in their affinity for the p75 neurotrophin receptor (p75^NTR^), whose expression is altered following SCI in the bladder and neuronal pathways governing micturition.^[102–104]^ Furthermore, proBDNF/proNGF preferentially activates the p75^NTR^-sortilin complex. Sortilin is a transport protein involved in the organization of intracellular cargo between membrane compartments. When activated by proneurotrophins in conjunction with p75^NTR^, it can induce apoptosis in many cell types.^[105]^ The mature neurotrophins activate their cognate Trk and p75^NTR^ heterodimer, promoting survival and differentiation.

The modulator of p75^NTR^, LM11A-31, mimics the binding activity of the loop one domain of NGF and competitively antagonizes proneurotrophin binding to the p75^NTR^-sortillin dimer.^[106]^ However, it can also potentially promote p75^NTR^-associated signaling through disinhibition and/or activation of the TrkA/B^NTR^-p75 complex.^[107]^ LM11A-31 reportedly ameliorates DSD and DO in SCI mice and also blocks the SCI-related urothelial damage and bladder wall remodeling.^[104,108]^ Thus, drugs targeting p75^NTR^ can modify DSD and/or DO in SCI and therefore assist in identifying underlying pathophysiological mechanisms and have therapeutic potential in SCI.

## Potential Targets for Detrusor-Sphincter Dyssynergia/Inefficient Voiding after Spinal Cord Injury

### Aδ-fiber-targeting treatment

**Figure 5:**
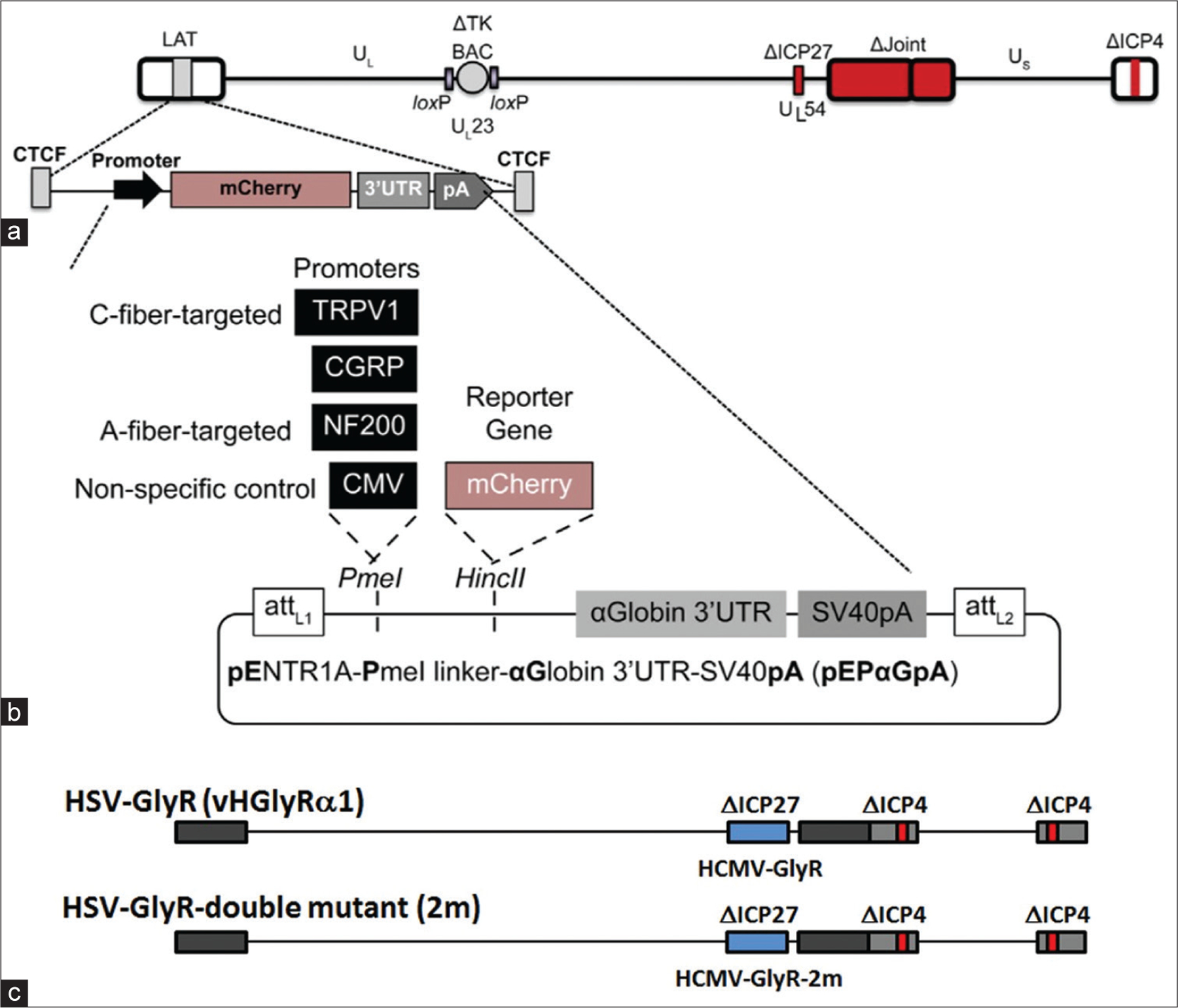
Schema of replication-deficient herpes simplex virus (HSV) vectors. (a) The vector backbone vLG is generated from the HSV KOS strain in which BAC sequences are inserted into the TK locus (UL23). Deletions are introduced in the internal repeat region and the genes encoding immediate early proteins ICP4 and ICP27, rendering the vector replication-defective. The Gateway destination cassette is inserted into the remaining latency locus, replacing the latency promoter elements while maintaining a wildtype copy of ICP0 and the surrounding CTCF chromatin boundary elements. (b) To generate afferent neuronal subtype-targeted expression vectors, transgenes are recombined into the vector backbone via the Gateway cassette. Individual promoter sequences (1284 bp TRPV1 promoter [TRPV1p], 932 bp CGRP promoter [CGRPp], 553 bp CMV promoter [CMVp], and 970 bp NF200 promoter [NF200p]) incorporating a Kozak consensus translation initiation sequence are inserted into the pENTR1A transfer vector, which contains attL sites for site-directed recombination with the attR-containing vector backbone. These Gateway plasmids are then recombined into the vLG vector backbone to generate the experimental vectors. (c) Schema of vectors. Replication-deficient HSV vectors in combination with wild type (upper) and the double mutant (G2M) (lower) α1 glycine receptor subunit gene. The study (Saito, *et al*., 2019^[107]^) utilized a chemogenetic approach using a G2M receptor with increased sensitivity to ivermectin, in combination with replication-defective HSV vector-mediated gene delivery methods. HSV: Herpes simplex virus

Ivermectin, which has been approved by the Food and Drug Administration for parasitic infections, activates a chloride flux in neurons expressing exogenous glutamate-gated chloride channel receptors (GluClRs), thereby shunting excitatory impulses causing hyperpolarization. Ivermectin-induced activation of mutant glycine receptors delivered to Aδ-fiber bladder afferents improves DSD and inefficient voiding in SCI mice, as shown before.^[109]^ The α1 glycine receptor subunit with double mutation (G2M) has a larger single-channel conductance, increasing the sensitivity to ivermectin almost 100-fold higher than wild-type glycine receptor. G2M can be delivered specifically to Aδ-fiber bladder afferent neurons by NF 200 (NF200) promoter-driven HSV vectors targeting NF-expressing Aδ-fiber neurons [Figure 5]. Histological studies show cell hypertrophy of Aδ-fiber bladder afferent neurons labeled by NF200 promoter-driven HSV vectors in SCI mice.^[109]^ Ivermectin-induced activation of G2M expressed in Aδ-fiber afferent neurons inoculation of NF200 promoter-driven HSV-G2M vectors into the bladder wall increases voiding efficiency and periods of reduced EUS-EMG activity during voiding bladder contractions.^[109]^ This also reduces the expression of Aδ-fiber-related mechanosensitive channels (ASICs, Piezo) in L6-S1 DRG, without altering the C-fiber-related channel (TRPV1) expression.^[109]^ Thus, the suppression of Aδ-fiber bladder afferent activity can potentially improve DSD and inefficient voiding in SCI [Figure 4].

### Piezo targeting treatment

Piezo1 and Piezo2, mechanosensitive ion channel proteins, are mainly expressed in the bladder and neurons, respectively. Piezo2 is mechanically activated and expressed in a subpopulation of sensory neurons, which is classified as low-threshold mechanosensory neurons (LTMR) that detect the direction of stimulus movements.^[110,111]^ A decrease in BDNF expression is associated with morphological polarization of Aδ-LTMR, leading to failure of direction-selective responses in these neurons.^[112,113]^ Piezo1 and Piezo2 channels are also expressed in the bladder epithelium and afferent pathways that can control low-threshold bladder-stretch sensing and micturition reflexes in mice.^[114,115]^

Time-course changes after SCI reveal that TRPV1 and ASIC1-3 on L6-S1 DRG are increased early during the 2–4 weeks’ period postinjury, but Piezo2 is increased in the later phase at 6 weeks’ postinjury.^[116]^ Intrathecal treatment of GxMTx4 (Piezo blocker) improves C-fiber-related bladder overactivity, the duration of EMG reduction time of the urethral sphincter, and voiding efficiency in SCI mice, as shown in our recent study.^[116]^ Thus, treatments targeting Piezo2 channels in afferent pathways might improve both storage and voiding dysfunction after SCI.

### Antifibrosis treatment

Urological diseases such as chronic bladder inflammation, benign prostate hyperplasia, and neurologic LUTD are often associated with bladder fibrosis.^[117–119]^ Transcripts of type 3 collagen, hypoxia-inducing factor-1α, transforming growth factor (TGF)-β1, and fibroblast growth factor (FGF) are increased in the bladder of SCI rats.^[120,121]^ Inefficient voiding due to DSD induces bladder distention, which evokes bladder ischemia, followed by bladder fibrosis.^[119]^ Bladder fibrosis with dense extracellular matrix deposition further deteriorates voiding dysfunction with reduced detrusor contractility, making the bladder stiffer, and less compliant.

Nintedanib, which inhibits the expression of fibrosis-related factor receptors including vascular endothelial growth factor (VEGF), FGF, and platelet-derived growth factor (PDGF), shows a lower risk of tumor promotion and is approved as a therapeutic agent for idiopathic pulmonary fibrosis patients.^[122]^ Our recent study reported that subcutaneous nintedanib treatment improves both storage and voiding dysfunctions, evident as increased voided volume per micturition and voiding efficiency in SCI mice.^[121]^ Nintedanib administration into SCI mice downregulates fibrosis-related molecules in the bladder including TGF-β1 and collagen type 1 and 3.^[121]^ Increased mRNA levels of TRPV1, TRPA1, P2X2, and P2X3 in lumbosacral DRG (C-fiber related) in SCI mice are significantly decreased, and the collagen deposition (trichrome staining) in the bladder is decreased after nintedanib treatment.^[121]^ Therefore, antifibrosis treatment using nintedanib, which inhibits VEGF, FGF, and PDGF receptors, can improve LUTD because of the bladder fibrosis in SCI. Furthermore, nintedanib-induced improvement of the bladder storage dysfunction might be mediated by modulation of bladder C-fiber afferent activity as seen by the downregulation of C-fiber afferent markers.

## Summary

**Figure 6:**
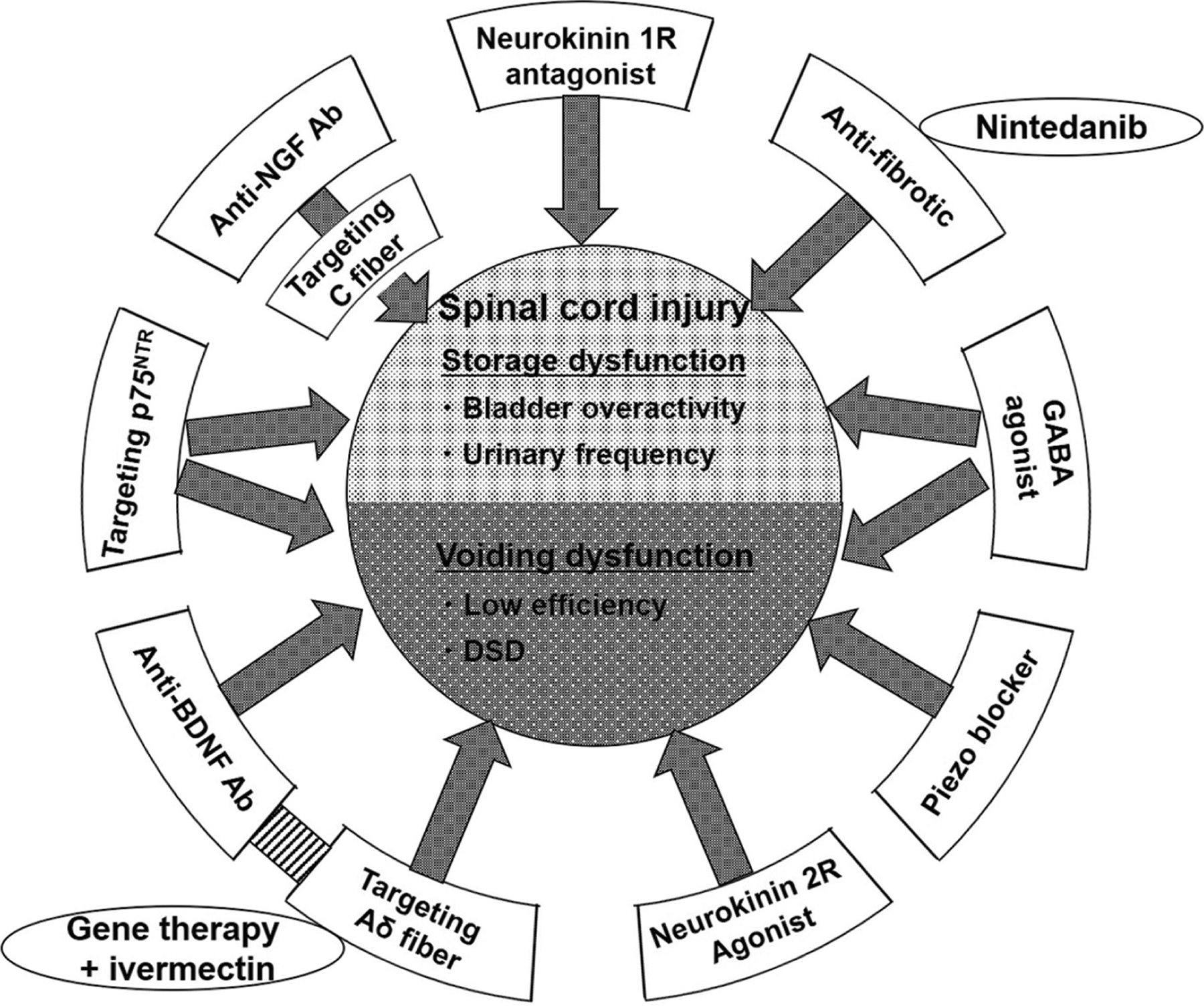
Summary of the potential targets for the treatments of LUTD after SCI. Several targets for the better/new treatments of LUTD due to SCI were reviewed in this article. Some targets are responsible for the storage dysfunction and others are for the voiding dysfunction such as DSD. These targets or medications need to be translated to the clinical setting. Some of them including ivermectin, nintedanib, mucimol or baclofen (GABA agonists) might be repositioned to new indications. LUTD: Lower urinary tract dysfunction, SCI: Spinal cord injury, DSD: Detrusor-sphincter dyssynergia

Various changes in the spinal/peripheral nervous systems and the LUT, including the bladder and the EUS seemingly contribute to neurogenic LUTD such as DO and DSD after SCI above the sacral level. Research using animal disease models is important to detect different pathophysiological mechanisms of LUTD. SCI animal studies indicate that the emergence of reflex bladder activity and development of neurogenic DO after SCI is partly due to plasticity in bladder C-fiber afferent pathways, which are silent under normal conditions. Furthermore, recent animal studies implicate that Aδ-fiber afferent pathways might be involved in the pathophysiology to induce LUTD such as DSD following SCI. The detection of different factors contributing to this neural plasticity might help uncover potential targets for better/new treatments of LUTD after SCI [Figure 6]. Further translational research based on data from animal studies is warranted for the future development of novel therapeutic modalities for treating SCI-induced LUTD.

## References

[1] Fowler CJ, Griffiths D, de Groat WC. The neural control of micturition. Nat Rev Neurosci 2008;9:453–66. 18490916 10.1038/nrn2401 PMC2897743

[2] de Groat WC, Griffiths D, Yoshimura N. Neural control of the lower urinary tract. Compr Physiol 2015;5:327–96. 25589273 10.1002/cphy.c130056 PMC4480926

[3] de Groat WC, Yoshimura N. Mechanisms underlying the recovery of lower urinary tract function following spinal cord injury. Prog Brain Res 2006;152:59–84. 16198694 10.1016/S0079-6123(05)52005-3

[4] de Groat WC, Yoshimura N. Afferent nerve regulation of bladder function in health and disease. Handb Exp Pharmacol 2009;194:91–138. 10.1007/978-3-540-79090-7_4 PMC3383010 19655106

[5] Birder L, Drake M, Groat WC, Fowler C, Mayer E, Morrison J, Neural control. In: Abrams P, Cardozo L, Khoury S, Wein A, editors. Incontinence Paris: Health Publication Ltd.; 2009. p. 169–253.

[6] Park JM, Bloom DA, McGuire EJ. The guarding reflex revisited. Br J Urol 1997;80:940–5. 9439415 10.1046/j.1464-410x.1997.00488.x

[7] Yoshimura N, de Groat WC. Neural control of the lower urinary tract. Int J Urol 1997;4:111–25. 9179682 10.1111/j.1442-2042.1997.tb00156.x

[8] Birder LA, de Groat WC, Apodaca G. Physiology of the urothelium. In: Shick E, Corcos J, editors. Textbook of the Neurogenic Bladder London, UK: Taylor and Francis; 2008. p. 19–39.

[9] Birder LA. Urothelial signaling. Auton Neurosci 2010;153:33–40. 19666243 10.1016/j.autneu.2009.07.005 PMC2818048

[10] Andersson KE, McCloskey KD. Lamina propria: The functional center of the bladder? Neurourol Urodyn 2014;33:9–16. 23847015 10.1002/nau.22465

[11] Birder LA, de Groat WC. Mechanisms of disease: Involvement of the urothelium in bladder dysfunction. Nat Clin Pract Urol 2007;4:46–54. 17211425 10.1038/ncpuro0672 PMC3119256

[12] de Groat WC. The urothelium in overactive bladder: Passive bystander or active participant? Urology 2004;64:7–11. 15621221 10.1016/j.urology.2004.08.063

[13] Ochodnicky P, Cruz CD, Yoshimura N, Cruz F. Neurotrophins as regulators of urinary bladder function. Nat Rev Urol 2012;9:628–37. 23045265 10.1038/nrurol.2012.178

[14] Fry CH, Kanai AH, Roosen A, Takeda M, Wood DN. Cell biology. In: Abrams P, Cardozo L, Khoury S, Wein A, ediotrs. Incontinence Paris: Health Publication Ltd.; 2009. p. 116–66.

[15] Cheng CL, de Groat WC. The role of capsaicin-sensitive afferent fibers in the lower urinary tract dysfunction induced by chronic spinal cord injury in rats. Exp Neurol 2004;187:445–54. 15144870 10.1016/j.expneurol.2004.02.014

[16] Kruse MN, Belton AL, de Groat WC. Changes in bladder and external urethral sphincter function after spinal cord injury in the rat. Am J Physiol 1993;264:R1157–63. 8322969 10.1152/ajpregu.1993.264.6.R1157

[17] Maggi CA, Giuliani S, Santicioli P, Meli A. Analysis of factors involved in determining urinary bladder voiding cycle in urethan-anesthetized rats. Am J Physiol 1986;251:R250–7. 3740305 10.1152/ajpregu.1986.251.2.R250

[18] Miyazato M, Sasatomi K, Hiragata S, Sugaya K, Chancellor MB, de Groat WC, Suppression of detrusor-sphincter dysynergia by GABA-receptor activation in the lumbosacral spinal cord in spinal cord-injured rats. Am J Physiol Regul Integr Comp Physiol 2008;295:R336–42. 18495826 10.1152/ajpregu.90315.2008 PMC2494799

[19] Kadekawa K, Yoshimura N, Majima T, Wada N, Shimizu T, Birder LA, Characterization of bladder and external urethral activity in mice with or without spinal cord injury – A comparison study with rats. Am J Physiol Regul Integr Comp Physiol 2016;310:R752–8. 26818058 10.1152/ajpregu.00450.2015 PMC4867409

[20] Yoshiyama M, deGroat WC, Fraser MO. Influences of external urethral sphincter relaxation induced by alpha-bungarotoxin, a neuromuscular junction blocking agent, on voiding dysfunction in the rat with spinal cord injury. Urology 2000;55:956–60. 10840125 10.1016/s0090-4295(00)00474-x

[21] Chang HY, Cheng CL, Chen JJ, de Groat WC. Serotonergic drugs and spinal cord transections indicate that different spinal circuits are involved in external urethral sphincter activity in rats. Am J Physiol Renal Physiol 2007;292:F1044–53. 17047164 10.1152/ajprenal.00175.2006 PMC3034291

[22] Karnup S. Spinal interneurons of the lower urinary tract circuits. Auton Neurosci 2021;235:102861. 34391124 10.1016/j.autneu.2021.102861 PMC8489359

[23] Karnup SV, de Groat WC. Propriospinal neurons of L3-L4 segments involved in control of the rat external urethral sphincter. Neuroscience 2020;425:12–28. 31785359 10.1016/j.neuroscience.2019.11.013 PMC6955099

[24] Karnup SV, De Groat WC. Mapping of spinal interneurons involved in regulation of the lower urinary tract in juvenile male rats. IBRO Rep 2020;9:115–31. 32775758 10.1016/j.ibror.2020.07.002 PMC7394742

[25] Somogyi GT, de Groat WC. Function, signal transduction mechanisms and plasticity of presynaptic muscarinic receptors in the urinary bladder. Life Sci 1999;64:411–8. 10069504 10.1016/s0024-3205(98)00580-3

[26] de Groat WC, Yoshimura N. Pharmacology of the lower urinary tract. Annu Rev Pharmacol Toxicol 2001;41:691–721. 11264473 10.1146/annurev.pharmtox.41.1.691

[27] Bennett BC, Kruse MN, Roppolo JR, Flood HD, Fraser M, de Groat WC. Neural control of urethral outlet activity in vivo: Role of nitric oxide. J Urol 1995;153:2004–9. 7752384

[28] Yono M, Yamamoto Y, Yoshida M, Ueda S, Latifpour J. Effects of doxazosin on blood flow and mRNA expression of nitric oxide synthase in the spontaneously hypertensive rat genitourinary tract. Life Sci 2007;81:218–22. 17574276 10.1016/j.lfs.2007.05.004 PMC2077832

[29] Thor KB, de Groat WC. Neural control of the female urethral and anal rhabdosphincters and pelvic floor muscles. Am J Physiol Regul Integr Comp Physiol 2010;299:R416–38. 20484700 10.1152/ajpregu.00111.2010 PMC2928615

[30] Andersson KE, Wein AJ. Pharmacology of the lower urinary tract: Basis for current and future treatments of urinary incontinence. Pharmacol Rev 2004;56:581–631. 15602011 10.1124/pr.56.4.4

[31] Hulsebosch CE, Coggeshall RE. An analysis of the axon populations in the nerves to the pelvic viscera in the rat. J Comp Neurol 1982;211:1–10. 7174880 10.1002/cne.902110102

[32] Uvelius B, Gabella G. The distribution of intramural nerves in urinary bladder after partial denervation in the female rat. Urol Res 1998;26:291–7. 9840337 10.1007/s002400050060

[33] Lawson SN, Perry MJ, Prabhakar E, McCarthy PW. Primary sensory neurones: Neurofilament, neuropeptides, and conduction velocity. Brain Res Bull 1993;30:239–43. 7681350 10.1016/0361-9230(93)90250-f

[34] Yoshimura N, Erdman SL, Snider MW, de Groat WC. Effects of spinal cord injury on neurofilament immunoreactivity and capsaicin sensitivity in rat dorsal root ganglion neurons innervating the urinary bladder. Neuroscience 1998;83:633–43. 9460769 10.1016/s0306-4522(97)00376-x

[35] Habler HJ, Janig W, Koltzenburg M. Activation of unmyelinated afferent fibres by mechanical stimuli and inflammation of the urinary bladder in the cat. J Physiol 1990;425:545–62. 2213588 10.1113/jphysiol.1990.sp018117 PMC1189862

[36] de Groat WC, Yoshimura N. Plasticity in reflex pathways to the lower urinary tract following spinal cord injury. Exp Neurol 2012;235:123–32. 21596038 10.1016/j.expneurol.2011.05.003 PMC3580860

[37] Kruse MN, Bennett B, De Groat WC. Effect of urinary diversion on the recovery of micturition reflexes after spinal cord injury in the rat. J Urol 1994;151:1088–91. 8126799 10.1016/s0022-5347(17)35189-3

[38] Ikeda Y, Fry C, Hayashi F, Stolz D, Griffiths D, Kanai A. Role of gap junctions in spontaneous activity of the rat bladder. Am J Physiol Renal Physiol 2007;293:F1018–25. 17581924 10.1152/ajprenal.00183.2007 PMC3037091

[39] Ikeda Y, Kanai A. Urotheliogenic modulation of intrinsic activity in spinal cord-transected rat bladders: Role of mucosal muscarinic receptors. Am J Physiol Renal Physiol 2008;295:F454–61. 18550643 10.1152/ajprenal.90315.2008 PMC2519189

[40] Kanai A, Roppolo J, Ikeda Y, Zabbarova I, Tai C, Birder L, Origin of spontaneous activity in neonatal and adult rat bladders and its enhancement by stretch and muscarinic agonists. Am J Physiol Renal Physiol 2007;292:F1065–72. 17107944 10.1152/ajprenal.00229.2006 PMC3033037

[41] Ng YK, de Groat WC, Wu HY. Smooth muscle and neural mechanisms contributing to the downregulation of neonatal rat spontaneous bladder contractions during postnatal development. Am J Physiol Regul Integr Comp Physiol 2007;292:R2100–12. 17234952 10.1152/ajpregu.00779.2006 PMC3111975

[42] Szell EA, Somogyi GT, de Groat WC, Szigeti GP. Developmental changes in spontaneous smooth muscle activity in the neonatal rat urinary bladder. Am J Physiol Regul Integr Comp Physiol 2003;285:R809–16. 12750150 10.1152/ajpregu.00641.2002

[43] Apodaca G, Kiss S, Ruiz W, Meyers S, Zeidel M, Birder L. Disruption of bladder epithelium barrier function after spinal cord injury. Am J Physiol Renal Physiol 2003;284:F966–76. 12527557 10.1152/ajprenal.00359.2002

[44] McCarthy CJ, Zabbarova IV, Brumovsky PR, Roppolo JR, Gebhart GF, Kanai AJ. Spontaneous contractions evoke afferent nerve firing in mouse bladders with detrusor overactivity. J Urol 2009;181:1459–66. 19157431 10.1016/j.juro.2008.10.139 PMC2899488

[45] Wada N, Shimizu T, Takai S, Shimizu N, Kanai AJ, Tyagi P, Post-injury bladder management strategy influences lower urinary tract dysfunction in the mouse model of spinal cord injury. Neurourol Urodyn 2017;36:1301–5. 27778376 10.1002/nau.23120 PMC5650194

[46] Yoshiyama M, de Groat WC. Effect of bilateral hypogastric nerve transection on voiding dysfunction in rats with spinal cord injury. Exp Neurol 2002;175:191–7. 12009771 10.1006/exnr.2002.7887

[47] Kadekawa K, Majima T, Kawamorita N, Okada H, Yoshizawa T, Mori K, Effects of an alpha1A/D-adrenoceptor antagonist, naftopidil, and a phosphodiesterase type 5 inhibitor, tadalafil, on urinary bladder remodeling in rats with spinal cord injury. Neurourol Urodyn 2017;36:1488–95. 27701772 10.1002/nau.23158

[48] Ishida H, Yamauchi H, Ito H, Akino H, Yokoyama O. α1D-Adrenoceptor blockade increases voiding efficiency by improving external urethral sphincter activity in rats with spinal cord injury. Am J Physiol Regul Integr Comp Physiol 2016;311:R971–8. 27605559 10.1152/ajpregu.00030.2016

[49] de Groat WC, Kawatani M, Hisamitsu T, Cheng CL, Ma CP, Thor K, Mechanisms underlying the recovery of urinary bladder function following spinal cord injury. J Auton Nerv Syst 1990;30 Suppl: S71–7. 10.1016/0165-1838(90)90105-r 2212495

[50] Cheng CL, Liu JC, Chang SY, Ma CP, de Groat WC. Effect of capsaicin on the micturition reflex in normal and chronic spinal cord-injured cats. Am J Physiol 1999;277:R786–94. 10484496 10.1152/ajpregu.1999.277.3.R786

[51] Cheng CL, Ma CP, de Groat WC. Effect of capsaicin on micturition and associated reflexes in chronic spinal rats. Brain Res 1995;678:40–8. 7620897 10.1016/0006-8993(95)00212-9

[52] Kadekawa K, Majima T, Shimizu T, Wada N, de Groat WC, Kanai AJ, The role of capsaicin-sensitive C-fiber afferent pathways in the control of micturition in spinal-intact and spinal cord-injured mice. Am J Physiol Renal Physiol 2017;313:F796–804. 28637786 10.1152/ajprenal.00097.2017 PMC5625111

[53] Brady CM, Apostolidis A, Yiangou Y, Baecker PA, Ford AP, Freeman A, P2X3-immunoreactive nerve fibres in neurogenic detrusor overactivity and the effect of intravesical resiniferatoxin. Eur Urol 2004;46:247–53. 15245821 10.1016/j.eururo.2003.12.017

[54] Brady CM, Apostolidis AN, Harper M, Yiangou Y, Beckett A, Jacques TS, Parallel changes in bladder suburothelial vanilloid receptor TRPV1 and pan-neuronal marker PGP9.5 immunoreactivity in patients with neurogenic detrusor overactivity after intravesical resiniferatoxin treatment. BJU Int 2004;93:770–6. 15049988 10.1111/j.1464-410X.2003.04722.x

[55] Apostolidis A, Fowler CJ. The use of botulinum neurotoxin type A (BoNTA) in urology. J Neural Transm 2008;115:593–605. 18322639 10.1007/s00702-007-0862-x

[56] Seki S, Erickson KA, Seki M, Nishizawa O, Igawa Y, Ogawa T, Elimination of rat spinal neurons expressing neurokinin 1 receptors reduces bladder overactivity and spinal c-fos expression induced by bladder irritation. Am J Physiol Renal Physiol 2005;288:F466–73. 15692058 10.1152/ajprenal.00274.2004

[57] Seki S, Erickson KE, Seki M, Nishizawa O, Chancellor MB, de Groat WC, Targeting spinal neurokinin I receptor-expressing neurons for the treatment of neurogenic detrusor overactiving in spinal cord injury. J Urol 2004;171:142–3.

[58] Zhang X, Douglas KL, Jin H, Eldaif BM, Nassar R, Fraser MO, Sprouting of substance P-expressing primary afferent central terminals and spinal micturition reflex NK1 receptor dependence after spinal cord injury. Am J Physiol Regul Integr Comp Physiol 2008;295:R2084–96. 18945947 10.1152/ajpregu.90653.2008 PMC2685299

[59] Burcher E, Shang F, Warner FJ, Du Q, Lubowski DZ, King DW, Tachykinin NK2 receptor and functional mechanisms in human colon: Changes with indomethacin and in diverticular disease and ulcerative colitis. J Pharmacol Exp Ther 2008;324:170–8. 17959748 10.1124/jpet.107.130385

[60] Mussap CJ, Stamatakos C, Burcher E. Radioligand binding, autoradiographic and functional studies demonstrate tachykinin NK-2 receptors in dog urinary bladder. J Pharmacol Exp Ther 1996;279:423–34. 8859022 10.1163/2211730x96x00225

[61] Warner FJ, Miller RC, Burcher E. Human tachykinin NK2 receptor: A comparative study of the colon and urinary bladder. Clin Exp Pharmacol. Physiol 2003;30:632–9. 12940880 10.1046/j.1440-1681.2003.03887.x

[62] Kullmann FA, Katofiasc M, Thor KB, Marson L. Pharmacodynamic evaluation of Lys(5), MeLeu(9), Nle(10)-NKA(4–10) prokinetic effects on bladder and colon activity in acute spinal cord transected and spinally intact rats. Naunyn-Schmiedebergs Arch Pharmacol 2017;390:163–73. 27889808 10.1007/s00210-016-1317-4 PMC5512890

[63] Marson L, Thor KB, Katofiasc M, Burgard EC, Rupniak NMJ. Prokinetic effects of neurokinin-2 receptor agonists on the bladder and rectum of rats with acute spinal cord transection. Eur J Pharmacol 2018;819:261–9. 29237540 10.1016/j.ejphar.2017.12.017 PMC5766413

[64] Rupniak NM, Katofiasc M, Walz A, Thor KB, Burgard EC.[Lys (5), MeLeu(9), Nle(10)]-NKA(4–10) elicits NK2 receptor-mediated micturition and defecation, and NK1 receptor-mediated emesis and hypotension, in conscious dogs. J Pharmacol Exp Ther 2018;366:136–44. 29728445 10.1124/jpet.118.248765

[65] Marson L, Piatt RK 2nd, Katofiasc MA, Bobbitt C, Thor KB. Chronic, twice-daily dosing of an NK2 receptor agonist [Lys5, MeLeu9, Nle10]-NKA(4–10), produces consistent drug-induced micturition and defecation in chronic spinal rats. J Neurotrauma 2020;37:868–76. 31642371 10.1089/neu.2019.6676 PMC7071061

[66] Miyazato M, Sugaya K, Nishijima S, Ashitomi K, Ohyama C, Ogawa Y. Rectal distention inhibits bladder activity via glycinergic and GABAergic mechanisms in rats. J Urol 2004;171:1353–6. 14767347 10.1097/01.ju.0000099840.09816.22

[67] Miyazato M, Sugaya K, Nishijima S, Ashitomi K, Hatano T, Ogawa Y. Inhibitory effect of intrathecal glycine on the micturition reflex in normal and spinal cord injury rats. Exp Neurol 2003;183:232–40. 12957506 10.1016/s0014-4886(03)00175-4

[68] Miyazato M, Sugaya K, Nishijima S, Ashitomi K, Morozumi M, Ogawa Y. Dietary glycine inhibits bladder activity in normal rats and rats with spinal cord injury. J Urol 2005;173:314–7. 15592103 10.1097/01.ju.0000141579.91638.a3

[69] Miyazato M, Sasatomi K, Hiragata S, Sugaya K, Chancellor MB, de Groat WC, GABA receptor activation in the lumbosacral spinal cord decreases detrusor overactivity in spinal cord injured rats. J Urol 2008;179:1178–83. 18206170 10.1016/j.juro.2007.10.030 PMC2744108

[70] Miyazato M, Sugaya K, Goins WF, Wolfe D, Goss JR, Chancellor MB, Herpes simplex virus vector-mediated gene delivery of glutamic acid decarboxylase reduces detrusor overactivity in spinal cord-injured rats. Gene Ther 2009;16:660–8. 19225548 10.1038/gt.2009.5 PMC2881227

[71] Miyazato M, Sugaya K, Saito S, Chancellor MB, Goins WF, Goss JR, Suppression of detrusor-sphincter dyssynergia by herpes simplex virus vector mediated gene delivery of glutamic acid decarboxylase in spinal cord injured rats. J Urol 2010;184:1204–10. 20663524 10.1016/j.juro.2010.04.066 PMC2921014

[72] Vizzard MA. Neurochemical plasticity and the role of neurotrophic factors in bladder reflex pathways after spinal cord injury. Prog Brain Res 2006;152:97–115. 16198696 10.1016/S0079-6123(05)52007-7

[73] Yoshimura N, Bennett NE, Hayashi Y, Ogawa T, Nishizawa O, Chancellor MB, Bladder overactivity and hyperexcitability of bladder afferent neurons after intrathecal delivery of nerve growth factor in rats. J Neurosci 2006;26:10847–55. 17050722 10.1523/JNEUROSCI.3023-06.2006 PMC6674760

[74] Keefe KM, Sheikh IS, Smith GM. Targeting Neurotrophins to Specific Populations of Neurons: NGF, BDNF, and NT-3 and Their Relevance for Treatment of Spinal Cord Injury. Int J Mol Sci 2017;18:E548. 28273811 10.3390/ijms18030548 PMC5372564

[75] Seki S, Sasaki K, Fraser MO, Igawa Y, Nishizawa O, Chancellor MB, Immunoneutralization of nerve growth factor in lumbosacral spinal cord reduces bladder hyperreflexia in spinal cord injured rats. J Urol 2002;168:2269–74. 12394773 10.1016/S0022-5347(05)64369-8

[76] Seki S, Sasaki K, Igawa Y, Nishizawa O, Chancellor MB, De Groat WC, Suppression of detrusor-sphincter dyssynergia by immunoneutralization of nerve growth factor in lumbosacral spinal cord in spinal cord injured rats. J Urol 2004;171:478–82. 14665959 10.1097/01.ju.0000088340.26588.74

[77] Wada N, Shimizu T, Shimizu N, de Groat WC, Kanai AJ, Tyagi P, The effect of neutralization of nerve growth factor (NGF) on bladder and urethral dysfunction in mice with spinal cord injury. Neurourol Urodyn 2018;37:1889–96. 29516546 10.1002/nau.23539 PMC6129225

[78] Cheng CL, Ma CP, de Groat WC. Effects of capsaicin on micturition and associated reflexes in the rat. Am J Physiol 1993;265:R132–8. 8342677 10.1152/ajpregu.1993.265.1.R132

[79] Seki S, Sasaki K, Igawa Y, Nishizawa O, Chancellor M, de Groat W, Detrusor overactivity induced by increased levels of nerve growth factor in bladder afferent pathways in rats. Neurourol Urodyn 2003;22:375–7.

[80] Steers WD, Tuttle JB. Mechanisms of disease: The role of nerve growth factor in the pathophysiology of bladder disorders. Nat Clin Pract Urol 2006;3:101–10. 16470209 10.1038/ncpuro0408

[81] Wada N, Matsumoto S, Kita M, Hashizume K, Kakizaki H. Decreased urinary nerve growth factor reflects prostatic volume reduction and relief of outlet obstruction in patients with benign prostatic enlargement treated with dutasteride. Int J Urol 2014;21:1258–62. 25039474 10.1111/iju.12570

[82] Chuang YC, Fraser MO, Yu Y, Chancellor MB, de Groat WC, Yoshimura N. The role of bladder afferent pathways in bladder hyperactivity induced by the intravesical administration of nerve growth factor. J Urol 2001;165:975–9. 11176525

[83] Lamb K, Gebhart GF, Bielefeldt K. Increased nerve growth factor expression triggers bladder overactivity. J Pain 2004;5:150–6. 15106127 10.1016/j.jpain.2004.01.001

[84] Vizzard MA. Changes in urinary bladder neurotrophic factor mRNA and NGF protein following urinary bladder dysfunction. Exp Neurol 2000;161:273–84. 10683293 10.1006/exnr.1999.7254

[85] Qiao L, Vizzard MA. Up‐regulation of tyrosine kinase (Trka, Trkb) receptor expression and phosphorylation in lumbosacral dorsal root ganglia after chronic spinal cord (T8‐T10) injury. J Comp Neurol 2002;449:217–30. 12115676 10.1002/cne.10283

[86] Ji RR, Samad TA, Jin SX, Schmoll R, Woolf CJ. p38 MAPK activation by NGF in primary sensory neurons after inflammation increases TRPV1 levels and maintains heat hyperalgesia. Neuron 2002;36:57–68. 12367506 10.1016/s0896-6273(02)00908-x

[87] Zhang X, Huang J, McNaughton PA. NGF rapidly increases membrane expression of TRPV1 heat-gated ion channels. EMBO J 2005;24:4211–23. 16319926 10.1038/sj.emboj.7600893 PMC1356334

[88] Shimizu N, Doyal MF, Goins WF, Kadekawa K, Wada N, Kanai AJ, Corrigendum to ‘Morphological changes in different populations of bladder afferent neurons detected by herpes simplex virus (HSV) vectors with Cell-type-specific promoters in mice with spinal cord injury’. Neuroscience 2017;364:190–201. 28942324 10.1016/j.neuroscience.2017.09.024 PMC5768486

[89] Andrade EL, Forner S, Bento AF, Leite DF, Dias MA, Leal PC, TRPA1 receptor modulation attenuates bladder overactivity induced by spinal cord injury. Am J Physiol Renal Physiol 2011;300:F1223–34. 21367919 10.1152/ajprenal.00535.2010

[90] Santos-Silva A, Charrua A, Cruz CD, Gharat L, Avelino A, Cruz F. Rat detrusor overactivity induced by chronic spinalization can be abolished by a transient receptor potential vanilloid 1 (TRPV1) antagonist. Auton Neurosci 2012;166:35–8. 22037502 10.1016/j.autneu.2011.09.005

[91] Wada N, Shimizu T, Shimizu N, Kurobe M, de Groat WC, Tyagi P, Therapeutic effects of inhibition of brain-derived neurotrophic factor on voiding dysfunction in mice with spinal cord injury. Am J Physiol Renal Physiol 2019;317:F1305–10. 31566429 10.1152/ajprenal.00239.2019

[92] Wada N, Yoshimura N, Kurobe M, Saito T, Tyagi P, Kakizaki H. The early, long-term inhibition of brain-derived neurotrophic factor improves voiding, and storage dysfunctions in mice with spinal cord injury. Neurourol Urodyn 2020;39:1345–54. 32394603 10.1002/nau.24385

[93] Garraway SM, Huie JR. Spinal plasticity and behavior: BDNF-induced neuromodulation in uninjured and injured spinal cord. Neural Plast 2016;2016:9857201. 27721996 10.1155/2016/9857201 PMC5046018

[94] Paddock N, Sheppard P, Gardiner P. Chronic increases in daily neuromuscular activity promote changes in gene expression in small and large dorsal root ganglion neurons in rat. Neuroscience 2018;388:171–80. 30031124 10.1016/j.neuroscience.2018.07.016

[95] Frias B, Santos J, Morgado M, Sousa MM, Gray SM, McCloskey KD, The role of brain-derived neurotrophic factor (BDNF) in the development of neurogenic detrusor overactivity (NDO). J Neurosci 2015;35:2146–60. 25653370 10.1523/JNEUROSCI.0373-14.2015 PMC4315839

[96] Mazzone GL, Veeraraghavan P, Gonzalez-Inchauspe C, Nistri A, Uchitel OD. ASIC channel inhibition enhances excitotoxic neuronal death in an in vitro model of spinal cord injury. Neuroscience 2017;343:398–410. 28003157 10.1016/j.neuroscience.2016.12.008

[97] McIlwrath SL, Hu J, Anirudhan G, Shin JB, Lewin GR. The sensory mechanotransduction ion channel ASIC2 (acid sensitive ion channel 2) is regulated by neurotrophin availability. Neuroscience 2005;131:499–511. 15708491 10.1016/j.neuroscience.2004.11.030

[98] Lewin GR, Moshourab R. Mechanosensation and pain. J Neurobiol 2004;61:30–44. 15362151 10.1002/neu.20078

[99] Dang K, Bielefeldt K, Gebhart GF. Differential responses of bladder lumbosacral and thoracolumbar dorsal root ganglion neurons to purinergic agonists, protons, and capsaicin. J Neurosci 2005;25:3973–84. 15829649 10.1523/JNEUROSCI.5239-04.2005 PMC6724937

[100] Lin SH, Cheng YR, Banks RW, Min MY, Bewick GS, Chen CC. Evidence for the involvement of ASIC3 in sensory mechanotransduction in proprioceptors. Nat Commun 2015;7:11460. 10.1038/ncomms11460 PMC4866049 27161260

[101] Lessmann V, Gottmann K, Malcangio M. Neurotrophin secretion: Current facts and future prospects. Prog Neurobiol 2003;69:341–74. 12787574 10.1016/s0301-0082(03)00019-4

[102] Teng KK, Felice S, Kim T, Hempstead BL. Understanding proneurotrophin actions: Recent advances and challenges. Dev Neurobiol 2010;70:350–9. 20186707 10.1002/dneu.20768 PMC3063094

[103] Vaidyanathan S, Krishnan KR, Mansour P, Soni BM, McDicken I. p75 nerve growth factor receptor in the vesical urothelium of patients with neuropathic bladder: An immunohistochemical study. Spinal Cord 1998;36:541–7. 9713922 10.1038/sj.sc.3100589

[104] Zabbarova IV, Ikeda Y, Carder EJ, Wipf P, Wolf-Johnston AS, Birder LA, Targeting p75 neurotrophin receptors ameliorates spinal cord injury-induced detrusor sphincter dyssynergia in mice. Neurourol Urodyn 2018;37:2452–61. 29806700 10.1002/nau.23722 PMC6202156

[105] Chao MV. Neurotrophins and their receptors: A convergence point for many signalling pathways. Nat Rev Neurosci 2003;4:299–309. 12671646 10.1038/nrn1078

[106] Hass MR, Sato C, Kopan R, Zhao G. Presenilin: RIP and beyond. Semin Cell Dev Biol 2009;20:201–10. 19073272 10.1016/j.semcdb.2008.11.014 PMC2693421

[107] Simmons DA, Belichenko NP, Ford EC, Semaan S, Monbureau M, Aiyaswamy S, A small molecule p75NTR ligand normalizes signalling and reduces Huntington’s disease phenotypes in R6/2 and BACHD mice. Hum Mol Genet 2016;25:4920–38. 28171570 10.1093/hmg/ddw316 PMC5418739

[108] Ryu JC, Tooke K, Malley SE, Soulas A, Weiss T, Ganesh N, Role of proNGF/p75 signaling in bladder dysfunction after spinal cord injury. J Clin Invest 2018;128:1772–86. 29584618 10.1172/JCI97837 PMC5919823

[109] Saito T Shimizu N, Goins W, Suzuki T, Shimizu T, Wada N, Ivermectin-induced activation of mutant glycine receptors delivered to Aδ-fiber bladder afferents by herpes simplex virus vectors driven by a subpopulation-specific neurofilament promoter improves detrusor-sphincter dyssynergia and inefficient voiding in mice with spinal cord injury. Neurourol Urodyn 2019;38 Suppl 3:S1–532.31350774

[110] Szczot M, Pogorzala LA, Solinski HJ, Young L, Yee P, Le Pichon CE, Cell-type-specific splicing of Piezo2 regulates mechanotransduction. Cell Rep 2017;21:2760–71. 29212024 10.1016/j.celrep.2017.11.035 PMC5741189

[111] Romero LO, Caires R, Nickolls AR, Chesler AT, Cordero-Morales JF, Vásquez V. A dietary fatty acid counteracts neuronal mechanical sensitization. Nat Commun 2020;11:2997. 32561714 10.1038/s41467-020-16816-2 PMC7305179

[112] Rutlin M, Ho CY, Abraira VE, Cassidy C, Bai L, Woodbury CJ, The cellular and molecular basis of direction selectivity of Aδ-LTMRs. Cell 2014;159:1640–51. 25525881 10.1016/j.cell.2014.11.038 PMC4297767

[113] Schrenk-Siemens K, Wende H, Prato V, Song K, Rostock C, Loewer A, PIEZO2 is required for mechanotransduction in human stem cell-derived touch receptors. Nat Neurosci 2015;18:10–6. 25469543 10.1038/nn.3894

[114] Marshall KL, Saade D, Ghitani N, Coombs AM, Szczot M, Keller J, PIEZO2 in sensory neurons and urothelial cells coordinates urination. Nature 2020;588:290–5. 33057202 10.1038/s41586-020-2830-7 PMC7725878

[115] Dalghi MG, Ruiz WG, Clayton DR, Montalbetti N, Daugherty SL, Beckel JM, Functional roles for PIEZO1 and PIEZO2 in urothelial mechanotransduction and lower urinary tract interoception. JCI Insight 2021;6:e152984. 34464353 10.1172/jci.insight.152984 PMC8525643

[116] Saito T, Gotoh D, Wada N, Tyagi P, Minagawa T, Ogawa T, Time-dependent progression of neurogenic lower urinary tract dysfunction after spinal cord injury in the mouse model. Am J Physiol Renal Physiol 2021;321:F26–32. 33969698 10.1152/ajprenal.00622.2020

[117] Azadzoi KM, Tarcan T, Kozlowski R, Krane RJ, Siroky MB. Overactivity and structural changes in the chronically ischemic bladder. J Urol 1999;162:1768–78. 10524933

[118] Iguchi N, Dönmez MI, Malykhina AP, Carrasco A Jr., Wilcox DT. Preventive effects of a HIF inhibitor, 17-DMAG, on partial bladder outlet obstruction-induced bladder dysfunction. Am J Physiol Renal Physiol 2017;313:F1149–60. 28768664 10.1152/ajprenal.00240.2017 PMC6148299

[119] Lee HJ, An J, Doo SW, Kim JH, Choi SS, Lee SR, Improvement in Spinal Cord Injury-Induced Bladder Fibrosis Using Mesenchymal Stem Cell Transplantation Into the Bladder Wall. Cell Transplant 2015;24:1253–63. 24912020 10.3727/096368914X682125

[120] Wada N, Shimizu T, Takai S, Shimizu N, Tyagi P, Kakizaki H, Combinational effects of muscarinic receptor inhibition and β3-adrenoceptor stimulation on neurogenic bladder dysfunction in rats with spinal cord injury. Neurourol Urodyn 2017b; 36:1039–45. 27367752 10.1002/nau.23066

[121] Kwon J, Lee EJ, Cho HJ, Jang JA, Han MS, Kwak E, Antifibrosis treatment by inhibition of VEGF, FGF, and PDGF receptors improves bladder wall remodeling and detrusor overactivity in association with modulation of C-fiber afferent activity in mice with spinal cord injury. Neurourol Urodyn 2021;40:1460–9. 34015154 10.1002/nau.24704

[122] Roth GJ, Binder R, Colbatzky F, Dallinger C, Schlenker-Herceg R, Hilberg F, Nintedanib: From discovery to the clinic. J Med Chem 2015;58:1053–63. 25474320 10.1021/jm501562a

